# Psychosocial adjustment to ALS: a longitudinal study

**DOI:** 10.3389/fpsyg.2015.01197

**Published:** 2015-09-14

**Authors:** Tamara Matuz, Niels Birbaumer, Martin Hautzinger, Andrea Kübler

**Affiliations:** ^1^Institute of Medical Psychology and Behavioral Neurobiology, Eberhard-Karls-University TübingenTübingen, Germany; ^2^Istituto di Ricovero e Cura a Carattere Scientifico, Ospedale San CamilloVenezia, Italy; ^3^Department of Psychology, Eberhard-Karls-University TübingenTübingen, Germany; ^4^Institute of Psychology, University of WürzburgGermany

**Keywords:** ALS, coping, depression, quality of life, longitudinal assessment

## Abstract

For the current study the Lazarian stress-coping theory and the appendant model of psychosocial adjustment to chronic illness and disabilities (Pakenham, 1999) has shaped the foundation for identifying determinants of adjustment to ALS. We aimed to investigate the evolution of psychosocial adjustment to ALS and to determine its long-term predictors. A longitudinal study design with four measurement time points was therefore, used to assess patients' quality of life, depression, and stress-coping model related aspects, such as illness characteristics, social support, cognitive appraisals, and coping strategies during a period of 2 years. Regression analyses revealed that 55% of the variance of severity of depressive symptoms and 47% of the variance in quality of life at T2 was accounted for by all the T1 predictor variables taken together. On the level of individual contributions, protective buffering, and appraisal of own coping potential accounted for a significant percentage in the variance in severity of depressive symptoms, whereas problem management coping strategies explained variance in quality of life scores. Illness characteristics at T2 did not explain any variance of both adjustment outcomes. Overall, the pattern of the longitudinal results indicated stable depressive symptoms and quality of life indices reflecting a successful adjustment to the disease across four measurement time points during a period of about two years. Empirical evidence is provided for the predictive value of social support, cognitive appraisals, and coping strategies, but not illness parameters such as severity and duration for adaptation to ALS. The current study contributes to a better conceptualization of adjustment, allowing us to provide evidence-based support beyond medical and physical intervention for people with ALS.

## Introduction

Being diagnosed with ALS constitutes for the afflicted person and its social environment a sudden and, considering the whole range of consequences, an unknown, critical life event. Life as known before the illness, with mostly stable social network, clearly defined life conceptions, wishes, plans, and expectations changes drastically. The impact of increasing physical impairment, due to gradual loss of motor functions, and the lack of a curative treatment specifically require substantial adaptive strategies from these patients. Against all odds and difficulties most of them report remarkable adjustment outcomes. Over the past two decades, several studies have shown that depression rate in the ALS population is surprisingly low (Kübler et al., 2005; Rabkin et al., 2005; Averill et al., 2007; Hammer et al., 2008; Lulé et al., 2008; Pagnini et al., 2015) and that good quality of life is maintained (Simmons et al., 2000; Goldstein et al., 2002; Nelson et al., 2003; Lulé et al., 2008, 2011). With few exceptions these studies focused on cross-sectional investigations. Most of the longitudinal studies on psychological variables in ALS assessed the various domains of patients' quality of life (Jenkinson et al., 1999; Goldstein et al., 2002, 2006; De Groot et al., 2007; Roach et al., 2009; Montel et al., 2012). All of these seem to agree that quality of life of ALS patients remains relatively constant. Some discrepancies can be observed regarding the relationship between QoL and severity of ALS, most reporting no significant associations (Simmons et al., 2000; Robbins et al., 2001; Goldstein et al., 2002; Lulé et al., 2008; Matuz et al., 2010; Gibbons et al., 2013) or only partially with specific QOL domains (McGuire et al., 1997; Jenkinson et al., 1999; Simmons et al., 2006). The evolution of indicators of mental health, such as depression and anxiety rates has been rarely addressed (Pagnini et al., 2015). The few studies doing so report a relatively high stability of mental health indices (Rabkin et al., 2005; Cupp et al., 2011; Montel et al., 2012; Lulé et al., 2014). However, there is a general problem regarding the assessment of prevalence of depression and anxiety in ALS. Estimation of the prevalence of depressive symptoms has been shown to be difficult (Pagnini, 2013). Determined by the employment of different instruments for the assessment of severity of depressive symptoms, different diagnostic interviews, sample size, and research question, a high variability of percentages reported across studies has been observed (Ferentinos et al., 2011; Pagnini et al., 2015). In an impressive meta-analysis Pagnini and colleagues showed that average scores for depressive symptoms as assessed by questionnaires such as BDI (Beck Depression inventory) or HADS (Hospital Anxiety and Depression Scale) are highly confounded with physical symptoms of the illness which are not necessarily related to depressiveness. They recommend the use of instruments specifically developed for people with ALS, such as the ADI-12 (Hammer et al., 2008) or the use of clinical interview in case of suspicion of a depressive disorder if the communication ability is still preserved (Pagnini et al., 2015).

Due to the fact that ALS patients are permanently challenged in different ways by the progression of their disease it seems reasonable to assess the evolution of their mental health, well-being and psychosocial adjustment indices to optimize support for coping with the disease.

**Figure 1:**
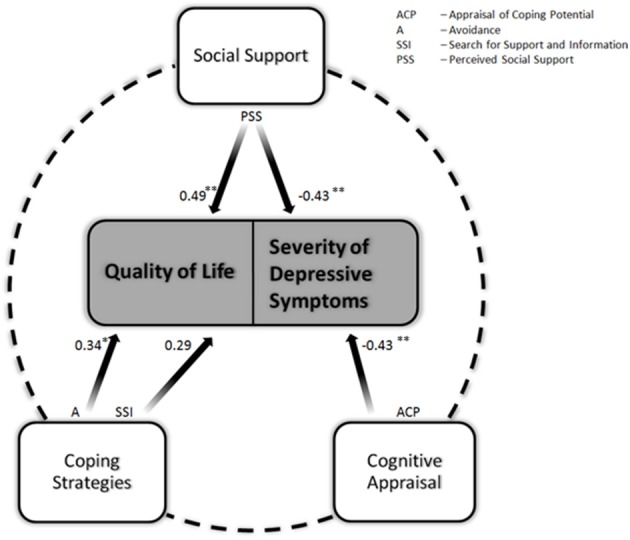
**The modified stress-coping model of psychosocial adjustment to ALS on the basis of the cross-sectional regression analyses at T1**.

Psychosocial adjustment to chronic illness and disabilities has been defined as a process in which patients have to adjust their internal needs to the new external demands such that they can achieve restoration of the person-environment equilibrium which is disrupted by the new life situation (Livneh and Antonak, 1997). Many factors have been considered when determining the psychosocial adjustment to chronic diseases (Stanton et al., 2007). For example, with respect to HIV and cancer, significant predictive relationships were found between psychosocial adjustment outcomes such as psychological distress and different type of coping strategies and the availability of social support (Stanton and Snider, 1993; Stanton et al., 2007). The model of psychosocial adjustment to chronic disease formulated by Pakenham and colleagues (Pakenham, 1999), being an adaptation of the stress-coping model proposed by Lazarus and Folkman (1984), provides an integrative framework of these factors. According to this, a complex interaction between *illness parameters, cognitive appraisal, coping resources*, and *coping strategies* determines the psychosocial adjustment to illness. Psychosocial adjustment can be operationalized through affective state and quality of life (Anderson, 2004). In our previous work in a group of 27 patients we applied this model and could identify predictors of psychosocial adjustment to ALS. We showed that social support, appraisal of coping potential and coping strategies such as independence, seeking for support and information and avoidance accounted for most of the variance in QoL, and depression scores (Figure 1). The severity of physical impairment and illness duration did not explain any variance in the adjustment outcomes (Matuz et al., 2010).

We continued our model driven research and aimed at assessing the determining variables over time. In the current study we examined weather stress-coping model variables found at T1 would also significantly predict depression and quality of life at T2. Moreover, we assessed the evolution of adjustment outcomes over four measurement time points and explored the changes of model variables over time. We hypothesized that—as for T1—the predicting factors would be cognitive appraisal, coping resources, and coping strategies, but not illness parameters.

## Materials and methods

Materials and Methods have been taken from our previous work (Matuz et al., 2010).

### Participants and procedure

The study was approved by the Ethical Committee of the Medical Faculty, University of Tübingen. The inclusion criterion for patients was a neurologist's diagnosis of ALS according to the El-Escorial criteria. Exclusion criteria were diagnosed frontotemporal dementia, alcoholism and poor knowledge of the German language. We used a longitudinal study design with four assessment time points (T1, T2, T3, and T4). The time between the assessments ranged between 3 and 6 months. Twenty-seven ALS patients (mean age ± *SD*: 55.3 ± 11.1; 35–73; 12 women) participated at T1. Twenty-two, nineteen, and sixteen patients were interviewed at T2, T3, and T4. Reasons for dropout included death (four patients) and deterioration of health (five patients). Two of the patients stated no reason for withdrawing their participation.

Patients were recruited from the ALS outpatient clinic of the Department of Neurology, University of Ulm, from the Institute of Medical Psychology and Behavioral Neurobiology, University of Tübingen and through an appeal for participation in the study in a biannual magazine published by the German Society for Muscular Diseases. All patients were visited at their homes by a clinical psychologist (TM), signed informed consent and were interviewed. Patients were interviewed alone, except those (*N* = 2 at T1 and T2, *N* = 3 at T3 and T4) who needed communicative support by caregivers or family members.

### Measures

#### Background data

Patients' demographic data referred to age, sex, marital status, level of education, and living arrangement and communication abilities, and were obtained with a semi-structured interview.

#### Illness parameters

Three illness related variables were assessed: *duration of illness* (month since diagnosis), *dependence on life sustaining treatment* (ventilation and nutrition), and *physical disability* [ALS Function Rating Scale (Cedarbaum et al., 1999) with scores range from 0 = complete paralysis to 40 = normal physical functioning].

#### Social support

The Berlin Social Support Scales (BSSS, Schulz and Schwarzer, 2003) was developed based on a multidimensional approach and includes five subscales measuring both cognitive and behavioral aspects of social support: perceived available social support, actually received social support, need for support, search for support, and protective buffering. Patients rated their agreement with the statements from each of the scales on a four-point Likert scale ranging from strongly disagree (1) to somewhat disagree (2), somewhat agree (3), and strongly agree (4). According to the users' manual the scales can be applied in conjunction or separately (Schulz and Schwarzer, 2003). To balance the predictors-sample size ratio we opted for using only the three following scales: perceived, received, and protective buffering.

#### Coping strategies

Coping strategies can be classified in four categories: *problem-management, problem-appraisal, emotion-management*, and *emotional-avoidance* (Terry and Hynes, 1998). Problem-management coping strategies describe active attempts to manage the situation (e.g., seek for information) whereas problem-appraisal coping involves attempts to avoid a direct and active confrontation with the problem by either reappraisals of the stressfulness of the situation (e.g., positive thinking) or by behavioral distraction (e.g., positive acting). Emotion-management strategies can be conceptualized as efforts to gain knowledge, to understand, and to express emotions engendered by the situation whereas emotional avoidance represents the active attempts to avoid emotions induced by the stressful events (Terry and Hynes, 1998; Osowiecki and Compas, 1999). Coping strategies were assessed with the Motor Neuron Disease Coping Scale (MNDCS) (Lee et al., 2001). Eighteen items are assigned in six subscales. Patients expressed the extent in which they relied on the coping strategies in the last month using a six-point Likert scale. We integrated the six subscales of this measure in the classification of coping strategies described above, as follows: information seeking and support scales as problem management strategies; positive thinking and positive action scales as problem appraisal strategies; independence scale as emotion management coping and avoidance as emotional avoidance strategy. One score for each type of coping was obtained by generating the mean score of the grouped scales.

#### Appraisal components

Patients' primary and secondary appraisals were assessed with four face-valid items designed to measure motivational relevance (“How important are the current events and illness related circumstances in your life?”), motivational congruence (“How congruent is your current life situation with the events and life circumstances you desired to experience?”), problem focused (“How confident are you in your abilities to maintain or change your life situation according to your wishes?”), and emotion focused coping potential (“How confident are you in your abilities to emotionally manage your current life situation?”). The items were originally provided by Smith and Lazarus (1993) and slightly modified to suit the current study population. Patients rated each item on a nine-point Likert scale (one referred to *not at all* and nine to *extremely*). Motivational relevance and congruence are the two components of the primary appraisal and combined appraisals of high relevance and and low congruence define the circumstances as stressful (Smith and Lazarus, 1993). For the analysis we used therefore difference scores of the two primary appraisal components and referred to as appraisal of threat. The two items assessing one's own coping potential (emotion and problem oriented coping) have been concatenated.

#### Depressive symptomatology

Severity of depressive symptoms was assessed with the ADI-12 (Hammer et al., 2008), an instrument specifically developed for the assessment of severity of depressive symptoms in ALS. Scores range from 12 to 48. We used a cut-off of ≥ 30 to identified patients with severe depressive symptoms, and a more liberal cut-off ≥ 23 to identify patients with any depressive disorder including minor depression. The instrument shows excellent internal consistency (Cronbach's α = 0.91, Hammer et al., 2008).

#### Individual quality of life

The schedule for the Evaluation of Individual Quality of Life-Direct Weighting (SEIQoL-DW) (Browne et al., 1997) allows the patients to propose five areas of their live which they consider most relevant for their QoL. They then indicate the relative importance of each area and the degree of satisfaction with each of the areas. The resulting SEIQoL index (see Results) ranges from 0 (worst possible QoL) to 100 (best possible QoL). According to a systematic review of 39 relevant studies in which SEIQoL-DW has been employed with a variety of populations, the instrument appears to be a feasible and valid one. Convergent and discriminant validity indices are ranging between moderate and strong (Wettergren et al., 2009).

### Statistical analysis

Normal distribution of the data was tested with Kolmogorov–Smirnov Test. We report the median (*Mdn*) when data were not normally distributed. On continuous data, correlational analyses were conducted to examine whether the dependent and independent variables varied as a function of the background data. Mann–Whitney *U*-test and Kruskal–Wallis *H*-test were applied to categorical data with the same purpose. Non-parametric tests were used with regard to the small sample size and when data were not normally distributed. To compare two variables within the same participants paired samples *t*-test or *Z* Wilcoxon signed-rank test were used. For the comparison of more than two conditions in the same participants, ANOVA or Friedman's ANOVA was computed. To evaluate the relative impact of the significant predictor variables at T1 on the measures of psychosocial adjustment at T2, two regression analyses were conducted separately for depression and QoL scores. This analysis could not be repeated for the rest of time points due to small sample sizes. For depression, the block of predictors included perceived social support, appraisal of coping potential, and emotion management coping (independence scale of the MNDCS). For QoL, we included perceived social support, problem-management coping strategies (information seeking and support), and avoidance. Since no hierarchical assumptions about these predictors were made, all of them were entered in one step using the method Enter. To investigate the relation between the aspects of the predictor variables and adjustment outcome at T2, cross sectional multiple regressions were performed on the dependent variables with the selected subscales of each stress-coping model predictor separately. The subscales on received social support, perceived social support, and protective buffering of BSSS were included in the regression analysis because these subscales are meant for the assessment of social support as a coping resource. The other scales of the instrument were related to coping behavior and, thus, were not included in the regression to avoid overlap with the coping strategy. From the items assessing cognitive appraisals we included primary appraisal of threat and appraisal of coping potential since these are central in the Lazarian stress-coping theory. The independent contribution of the subscales to the prediction of the dependent variable was considered significant only after Bonferroni correction (at *p* < 0.01). Analysis of the model assumptions (i.e., independence of the residuals, absence of outliers, linearity and homoscedasticity) was performed. We used SPSS 15.0 for all statistical analyses.

## Results

Most of the patients had spinal onset and sporadic form of ALS. About half of the sample was artificially ventilated (either by mask or through tracheostoma) and approximatively one quarter of the interviewed patients were fed via PEG. The lower/higher education ratio as well as female/male ratio was well-balanced in the current sample. All background and disease related data are listed in Table 1.

**Table 1:** **Background information**.

	Education^*^		**Sex**		**Marital status**				**Artificial ventilation**		**PEG**	
	**Lower**	**Higher**	**M**	**F**	**Married**	**Single**	**Widowed**	**Divorced**	**Yes**	**No**	**Yes**	**No**
T1	48.1	51.9	55.6	44.4	77.8	11.1	7.4	3.7	44.4	55.6	22.2	77.8
T2	45.5	54.5	54.5	45.5	77.3	13.6	9.1	–	40.9	59.1	27.3	72.7
T3	42.1	57.9	63.2	36.8	84.2	15.8	–	–	42.2	57.8	42.1	57.9
T4	37.5	62.5	62.5	37.8	81.3	18.7	–	–	56.3	43.7	37.5	62.5
	**Type of ALS**		**Onset of ALS**		Communication^****^				**Living environment**			
	Spor.^**^	Gen.^**^	B^***^	S^***^	**Normal**	**Impaired**		**Assisted by device**	**Own household**		**Nursing home**	
T1	100	0	7.4	92.6	26	55.5		18.5	92.6		7.4	
T2	100	0	4.6	95.4	27.3	31.8		40.9	90.9		9.1	
T3	100	0	5.3	94.7	26.3	26.3		47.4	94.7		5.3	
T4	100	0	0	100	31.3	25		43.7	93.7		6.3	

* *Lower education refers to 10 years of school (secondary school, first phase) with or without vocational training. Higher education refers to a duration longer than 10 years*.

** *Spor., Sporadic; Gen., Genetic*.

*** *B, Bulbar; S, Spinal*.

**** *Normal communication refers to normal speech process; impaired communication ranged from “Detectable speech disturbance” to “communication intelligible with repeating”; assisted by devices refers to loss of useful speech and communication exclusively possible by using assistive technology and/or eye movements*.

### Psychosocial adjustment outcomes and demographics

**Figure 2:**
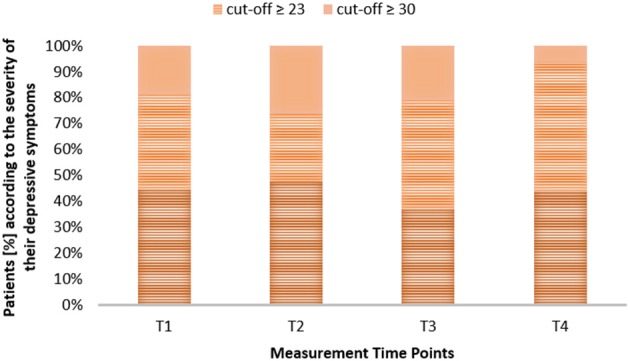
**Distribution of the patients depending on the severity of their symptoms over the measurement time points**.

The group's ADI-12 mean scores are summarized in Table 2. The distribution of the patients (%) depending on the severity of their symptoms (not depressed, presence of any depressive disorder and major depression) are depicted in Figure 2. Mean scores of individual QoL are reported in Table 2.

**Table 2:** **Descriptives for depression and quality of life over the measurement time points**.

**Assessment time point**	**T1**		**T2**		**T3**		**T4**	
**Sample size**	***N*** = 27		***N* = 21**	***N* = 22**	***N* = 18**	***N* = 19**	***N* = 15**	***N* = 16**
Outcome	QoL	D	QoL	D	QoL	D	QoL	D
Mean/Mdn	67.79	22.5	66.67	23	67.56	21.53	67.47	21.19
*SD*	15.85	6.71	16.26	6.85	16.27	6.42	14.86	4.65
Range	32–91	12–36	37–94	12–39	42–96	12–34	34–86	12–32

*QoL, Quality of Life; D, Depression*.

There were no significant differences between the mean scores of the four measurements for both adjustment outcomes. Depression and quality of life mean scores remained constant over the measurement time points [ADI-12: $\chi_{\left(2,\;N=22\right)}^{2}=4.64$, *p* < 0.2, Friedman Test; SEIQoL: *F*(1, 14) = 0.61, *p* < 0.4, repeated measures ANOVA]. Pairwise comparisons revealed however significantly lower ADI-12 scores at T4 (*Mdn* 20) than at T1 (*Mdn* 22.5) (*z* = −2.4, *p* < 0.05, Wilkoxon rank Test).

For each of the measurement time points we investigated the relationship between demographical aspects and adjustment outcomes. Age correlated significantly positive with QoL at T1 and T3 (T1: *r* = 0.49, *p* < 0.01; T3: *r* = 0.51, *p* < 0.05; Pearsons Correlations). No significant correlations were found between depression and age. For each of the measurement time points both depression and QoL did not differ between sexes and as a function of education (for statistical coefficients see Table 1 of Supplementary Materials).

### Predictors of psychosocial adjutment: descriptives and longitudinal analysis

With mean scores ranging between 17.4 and 13.2 ALS-FRS values indicated high physical impairment of the patient sample. Average scores of ALS FRS and the illness duration (months since trying to spare their partners negative information and displaying strength and general good mood diagnosis) are summarized in Table 3. The average severity of the physical impairment increased significantly over time reflecting the degenerative character of the disease [$\chi_{\left(3,\;N=22\right)}^{2}=13.08$, *p* < 0.01, Friedman Test].

**Table 3:** **Mean scores of model variables**.

		**T1**	**T2**	**T3**	**T4**
**ILLNESS PARAMETERS**					
ALS FRS	M/Mdn	17.4	15.9	14.4	13.2
	*SD*	9.8	10.1	10.1	11.1
	Range	0–36	0–36	0–35	0–34
	Maximum possible	40	40	40	40
Illness duration	M/Mdn	43.2	40.9	58.1	63.2
	*SD*	30.5	31.6	33.3	32.1
	Range	4–129	18–131	22–135	26–141
**BSSS SCALES**					
Perceived support	M/Mdn	29.6	28	29	29.3
	*SD*	3.9	5.5	3.8	3.4
	Range	17–32	14–32	19–32	22–31
	Maximum possible	32	32	32	32
Received support	M/Mdn	41.3	40	41.4	44.5
	*SD*	8.8	10.3	9.1	5.4
	Range	23–48	15–48	12-48	30-48
	Maximum possible	60	60	60	60
Search for support	M/Mdn	14.4	14.3	14.2	15.1
	*SD*	3.1	3.2	3.4	4.4
	Range	6-20	6-20	5-20	3-20
	Maximum possible	20	20	20	20
Need for support	M/Mdn	11.8	10.1	9.8	10.2
	*SD*	2.9	1.6	2.1	2.4
	Range	5-16	6-13	6-14	6-14
	Maximum possible	16	16	16	16
Protective buffering	M/Mdn	13.4	13.4	14.2	13.8
	*SD*	4.1	3.2	4.5	4.2
	Range	7-21	7-20	6-24	7-20
	Maximum possible	24	24	24	24
**COPING STRATEGIES**					
Problem management	M/Mdn	24.6	22.2	22.7	23.7
	*SD*	4.4	4.7	4.1	4.3
	Range	13-30	12-29	15-30	18-30
	Maximum possible	30	30	30	30
Problem appraisal	M/Mdn	20	18.9	18.5	20.3
	*SD*	4.6	5.2	3.1	2.9
	Range	10-25	7-25	12–23	14–23
	Maximum possible	25	25	25	25
Emotion management	M/Mdn	18	17.3	16.8	18.2
	*SD*	1.8	2.6	2.4	1.6
	Range	14–20	12–20	11–20	15–20
	Maximum possible	20	20	20	20
Emotional avoidance	M/Mdn	6.9	6	6.6	6
	*SD*	2.4	2.4	2.8	3.9
	Range	2–12	1–10	2–13	0–15
	Maximum possible	15	15	15	15
**COGNITIVE APPRAISALS**					
Appraisal of threat	M/Mdn	3.6	3.3	1.2	3.4
	*SD*	2.1	2.1	1.3	2.3
	Range	0–8	0–8	0–4	0–7
	Maximum possible	8	8	8	8
Appraisal of coping potential	M/Mdn	5.83	5.91	5.3	6.5
	*SD*	1.7	1.7	1.9	1.4
	Range	2.5–9	2.5–8	2–8	4.5–9
	Maximum possible	9	9	9	9
Self-accountability	M/Mdn	1	1	4.9	1
	*SD*			2.04	
	Range	1–9	1–8	1–9	1–7
	Maximum possible	9	9	9	9
Other accountability	M/Mdn	1	1.5	2	1
	*SD*				
	Range	1–7	1–6	1–9	1–9
	Maximum possible	9	9	9	9
Future expectancy	M/Mdn	4.5	5.3	2	4.8
	*SD*	2.3	2.4		2.8
	Range	1–9	1–9	1–9	1–9
	Maximum possible	9	9	9	9

Scores of received and perceived social support indicated a high and constant availability of social support in this sample (Table 3). No significant differences were found between the four time points [for perceived: $\chi_{\left(3,\;N=22\right)}^{2}=1.4$, *p* = 0.7; for received: $\chi_{\left(3,\;N=22\right)}^{2}=1.6$, *p* = 0.6; Friedman Tests]. Cohabitating patients showed on average high protective behavior by trying to spare their partners negative information and displaying strength and general good mood. Scores of protective buffering scale did not change over time [$\chi_{\left(3,\;N=22\right)}^{2}=2.8$, *p* = 0.4; Friedman Test].

With respect to coping strategies, Friedman's ANOVA revealed significant differences between the frequencies of problem- and emotion-management strategies over the measurement time points. Wilcoxon signed-rank tests were used for pairwise comparisons (Bonferroni corrected) and indicated that, patients relied more frequently on both problem- and emotion- management strategies at T1 compared to the other time points. Whereas at T3 patients used less frequently emotion-management strategies. Scores of problem-appraisal and avoidant coping remained stable over time [for independence: χ2_(3, *N* = 22)_ = 5.75, *p* = 0.1; for avoidance: $\chi_{\left(3,\;N=22\right)}^{2}=0.76$, *p* = 0.9; Friedman Tests].

Mean scores of primary appraisals at all the measurement time points reflected that patients rated their illness situation as being rather threatening at T1, T2, and T4 (Table 3). Scores at T3 were significantly lower compared to all the other time points [$\chi_{\left(3,\;N=22\right)}^{2}=7.6$, *p* = 0.05; Friedman Test], indicating that patients believed at this time point that their illness situation is not threatening at all. Moreover, patients constantly rated their own coping potentials as being high (for mean values see Table 3). This experienced high propensity to cope well with their disease did not change over time [$\chi_{\left(3,\;N=22\right)}^{2}=4.9$, *p* = 0.1; Friedman Test].

At T1 significant mean differences were found between the appraisals of problem-focused coping potential and emotion-focused coping potential [*t*_(26)_ = −4.5, *p* < 0.001, Paired sample *t*-test], indicating that patients believed to deal better with the situation emotionally than by active attempts to change it. However, this difference could not be found for the other time points.

### Regression analyses

**Figure 3:**
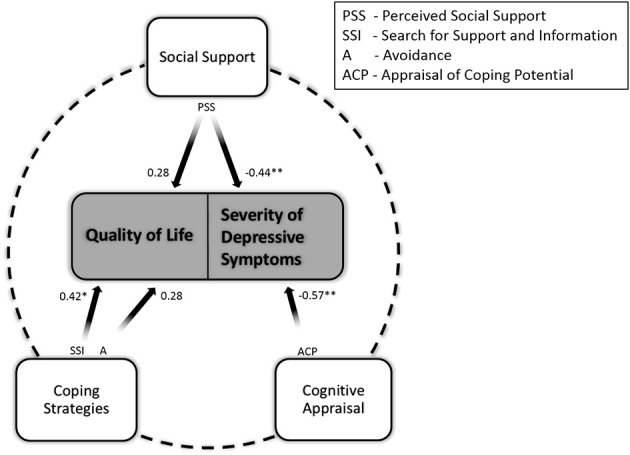
**The modified stress-coping model of psychosocial adjustment to amyotrophic lateral sclerosis on the basis of longitudinal regression analyses**. The psychosocial predictor variables at T1 accounted together for a considerable amount of variance in both outcomes of psychosocial adjustment (depression and quality of life) at T2. This is illustrated by the dashed line. Variables that displayed independent predictive power are denoted by stars.

A regression was conducted to determine whether stress-coping model variables that were identified at T1 as being significant predictors continued to predict depression and respectively quality of life at T2. Results showed that 55% of the variance of severity of depressive symptoms at T2 was accounted for by all the T1 predictor variables taken together. The *b*-values (Table 4) indicated the independent contribution of each predictor to the total explained variance. Hence, perceived social support and appraisal of own coping potential individually accounted for a significant percentage in the variance in severity of depressive symptoms. Higher perceived social support [*t*_(22)_ = 2.28; *p* < 0.05] and more confidence in their own coping potential [*t*_(22)_ = 2.49; *p* < 0.05] predicted lower depressed mood. Similarly to T1, none of the coping strategies predicted the severity of depressive symptoms. Predictors that were selected at T1 continued to explain 47% of the variance in QoL at T2 [*F*_(3, 23)_= 5.01; *p* < 0.01]. On the level of individual contributions, *b*-values indicated only for problem management coping strategies significant results. Patients who searched more frequently for information and support reported higher QoL. Results are depicted in Figure 3.

**Table 4:** **Summary of regression analysis of stress-coping variables at T1 predicting psychological adjustment to amyotrophic lateral sclerosis at T2**.

**Predictors**	**Dependent variables**	**Depression**			**Quality of life**		
		**B**	**SE B**	**ß**	**B**	**SE B**	**ß**
Social support	Perceived social support	**−0.95**	**0.39**	−0.44^**^	1.7	1.11	0.28
Cognitive appraisal	Appraisal of coping potential	**−2.30**	**0.69**	−0.57^**^			
Coping strategies	Problem management				**3.2**	**1.45**	0.42^*^
	Emotion management	0.09	0.69	0.02			
	Emotional avoidance				1.79	1.17	0.28
		*R*² = 0.55, Δ*R*² = 0.47 ***F*_(3, 23)_ = 7.2, *p* = 0.002**			*R*² = 0.47, Δ*R*² = 0.37 ***F*_(3, 23)_ = 5.01, *p* = 0.01**		

*Bold values indicate statistically significant contribution in explaining the variance*.

* p < 0.01;

** *p < 0.05*.

Cross-sectional regression analyses revealed that at T2 neither severity of depressive symptoms nor QoL could be predicted by the degree of physical impairment and illness duration. A summary of the regression analyses is presented in Table 5. Depression was significantly predicted by cognitive appraisals. Appraisal of own coping potential significantly contributed to explaining portions of the total variance of depression scores, higher appraisal of own coping potential predicting lower severity of depressive symptoms. The variance of depression scores could not be significantly accounted for by any of the coping and social support scales. On the contrary, higher social support significantly predicted higher quality of life values. The independent contribution of protective buffering scale to the total explained variance of the quality of life scores was significant. Coping strategies did account for a significant amount of the variance of quality of life, with problem management strategies (seek for information and support) being most predictive. However, this relation did not reach significance at the corrected *p*-value. Likewise, none of the appraisal scales could significantly predict quality of life.

**Table 5:** **Summary of regression analyses on the two adjustment variables with the subscales of each stress–coping predictors separately at T2**.

**Predictors**	**Dependent variables**	**Depression**			**Quality of life**		
		**B**	**SE B**	**ß**	**B**	**SE B**	**ß**
Social support	Perceived	−0.65	0.41	−0.51	1.08	1.12	0.30
	Received	0.12	.22	−0.17	0.85	0.48	0.53
	Protective buffering	**1.14**	**0.46**	0.53^*^	**−2.27**	**0.96**	−0.46^*^
		*R*² = 0.32, Δ*R*² = 0.20 ***F*_(3, 17)_ = 2.7, *p* = 0.07**			*R*² = 0.45, Δ*R*² = 0.35 ***F*_(3, 17)_ = 6.2, *p* = 0.005**		
Cognitive appraisal	Primary appraisal	−0.48	0.62	−0.15	0.26	1.92	0.03
	Appraisal of coping potential	**−2.84**	**0.78**	−0.69^**^	3.89	2.67	0.38
		*R*² = 0.42, Δ*R*² = 0.36 ***F*_(2, 19)_ = 6.9, *p* = 0.006**			*R*² = 0.13, Δ*R*² = 0.03 *F*_(2, 18)_ = 1.3, *p* = 0.2		
Coping strategies	Problem management	0.42	0.80	0.14	3.5	1.87	0.48
	Problem appraisal	−1.17	1.05	−0.44	−0.65	2.38	−0.09
	Emotion management	−0.64	0.98	−0.24	2.22	2.14	0.35
	Emotional avoidance	0.003	0.74	0.001	−0.85	1.67	−0.13
		*R*² = 0.38, Δ*R*² = 0.24 *F*_(4, 17)_ = 2.6, *p* = 0.07			*R*² = 0.46, Δ*R*² = 0.33 ***F*_(4, 16)_ = 3.4, *p* = 0.03**		
Illness parameters	Physical impairment	0.05	0.16	0.08	−0.21	0.39	−0.13
	Time since diagnosis	0.02	0.05	0.12	0.07	0.13	0.13
		*R*² = 0.01, Δ*R*² = −0.08 *F*_(2, 19)_ = 0.14, *p* = 0.8			*R*² = 0.04, Δ*R*² = −0.06 F_(2, 18)_ = 0.37, *p* = 0.7		

*Bold values indicate statistically significant contribution in explaining the variance*.

* p < 0.01;

** *p < 0.05*.

## Discussion

### Short and long-term psychosocial adjustment to ALS

For our longitudinal study the adaptation of Lazarian stress-coping theory to chronic illness and disabilities (Pakenham, 1999) has shaped the foundation for identifying determinants of adjustment to ALS. Longitudinal regression analysis showed that significant predictors of adjustment outcomes, as found at T1 (Matuz et al., 2010), continued to predict to a large extent adjustment at T2. Perceived social support together with appraisal of one's own coping potential and independence explained 55% of the variance of severity of depressive symptoms. Perceived social support, seek for information and support and avoidance accounted for 47% of the variance in quality of life indices.

We characterized predictive factors for favorable adjustment to ALS over time. The pattern of the longitudinal results indicated stable depressive symptoms and quality of life indices reflecting a successful adjustment to the disease across four measurement time points during a period of about 2 years. Although the sample reported on average low depressive symptoms and high quality of life, considerable variability in the severity of depressive symptoms and quality of life scores across persons and time was apparent. These results are in line with the existing evidences from previous research on psychological aspects in ALS (Rabkin et al., 2005, 2015; Lulé et al., 2008; McElhiney et al., 2009). Several studies showed that many individuals with ALS report good adjustment, however, evidence for heterogeneity in trajectories of adjustment across individuals and time have also been shown (Pagnini, 2013). Differences between depression scores at T1 and T4 in our sample suggest that people with ALS can eventually hone their adjustment over time. The mechanisms which are responsible for this improvement are still not clear, however, it is possible that people with ALS apply strategies as described by the so called TOTE model (Miller et al., 1986). According to this, individuals first test for the presence of a difference between their expectations and their current experiences and then engage in various actions to reduce this difference. Coping strategies are here important mediators. The difference is then tested again, and the cycle iterates until expectations and experiences match. For the case of people with ALS it has been previously argued that as the disease progresses patients might reshape and redefine their expectations so that these match with their ongoing experience and thus leading to less negative feelings and higher quality of life (Real et al., 2014). Future research is nevertheless needed to address this issue explicitly for example by clustering and characterizing adaptors and non-adaptors across time.

On the level of individual contributions we found that higher perceived social support and appraisal of own coping potential continued to significantly predict lower depressive symptoms. Likewise, more frequent use of problem management coping strategies, such as seeking for support and information continued to predict higher quality of life.

Social support has been shown to affect adaptive outcomes through cognitive, emotional, and physiological pathways (Wills and Fegan, 2001). A supportive, uncritical social environment can help patients use effective coping strategies, encourage positive health behaviors, and diminish physiological reactivity to stress. For ALS, research has repeatedly underlined the importance of social networks, familiar support, and availability of technological aid devices (Chió et al., 2004; Goldstein et al., 2006; McLeod and Clarke, 2007; Olsson et al., 2010; Tramonti et al., 2012). Our results indicate that perceived social support, which reflects patients view about the quantity and quality of the support received, successfully predicted depressive symptoms over time. Severity of depressive symptoms can be obviously well-predicted in ALS by cognitive appraisal processes. Our results further support this statement by showing that patients perceiving their own coping potential as high experienced lower severity of depressive symptoms across time points.

Similar to our previous outcomes of the cross sectional analysis (Matuz et al., 2010) revealed that none of the coping strategies did predict severity of depressive symptoms over time. Coping had rather an effect on quality of life. Search for information and support significantly predicted high quality of life over time. Problem focused coping attempts have been repeatedly reported as being more adaptive for patients with chronic diseases (Young, 1992; Keefe et al., 2002; Savelkoul et al., 2003) and to some extent for ALS patients as well (Montel et al., 2012).

Avoidant coping has on the contrary lost its predictive power for quality of life at T2. Coping strategies people employ and their utility are likely to vary as the adaptive tasks of illness change (Blalock et al., 1993). For example, breast cancer patients who reported frequent use of cognitive avoidance prior to breast biopsy showed less distress at that point, however, higher distress scores after cancer diagnosis and after surgery (Stanton and Snider, 1993; Lutgendorf et al., 2002; Hack and Degner, 2004). Avoidant coping strategies may be useful at acute points of specific crisis in the progress of the illness, however, over time avoidance is typically related to maladjustment. Based on our results this seems to be true also for ALS. It may prevent them from taking measures necessary for coping with the disease in the future, such as seeking information about assistive technology for communication or artificial nutrition.

Unexpectedly, perceived social support did not continue to predict quality of life at T2. Consequences of chronic disease can be abrupt and distinctive (e.g., surgeries, progression of specific symptoms), or they can be rather gradual (e.g., declines in vitality, social contacts) (Thompson and Kyle, 2000). Changes in patients' social relations and networks can proceed with an uneven course (Stanton et al., 2007) shaping the interaction between social support and adjustment outcomes. The current cross-sectional results at T2 support this notion by revealing significant contribution of social support in explaining the variance of quality of life indices, yet this time by the individual contribution of the subscale protective buffering. Thus, people with ALS manifesting more protective behavior toward their partners, e.g., withholding bad news, showing more strength experience higher quality of life. Whereas partners' overprotection (including protective buffering) is considered an unsupportive behavior (Schokker et al., 2010; Vilchinsky et al., 2011), patients' protective buffering acts toward their partners and their positive effects on adjustment received little attention in the literature. Being more protective with their partners presumably helps ALS patients in reducing their own perception of being a burden and eventually increases their perception of self-efficacy.

Last but not least, cross sectional regression revealed that none of the adjustment outcomes could be significantly predicted by illness parameters. Severe functional impairment as well as ALS duration are not necessarily related to poorer quality of life and depressive mood. With these results we contribute to the still ongoing debate among ALS care specialists over the role of severity of physical impairment and illness related characteristics in maintaining mental health and quality of life in people with ALS. Our both cross sectional and longitudinal findings indicate that psychosocial aspects are better predictors of adjustment to ALS than illness related characteristics.

### Implications for clinical and home healthcare

Overall, the analyses presented here extend the literature on psychological aspects in ALS and can guide psychological interventions developed for people with ALS.

Although mainly limited by the sample size and the drop outs due to death and illness progression, our results enriched the understanding of psychosocial adjustment to ALS. Empirical evidence is provided for the predictive utility of the adaptation of the stress-coping model in finding those factors that promote or hinder psychosocial adjustment to ALS. Also, the current study contributes to a better conceptualization of adjustment, showing that ALS necessitates adjustment in multiple life domains as the diseases advances.

Existing psychological interventions in ALS can be rendered more efficacious by including the current findings. Whereas cognitive therapeutic strategies targeting appraisal of coping potential and available social resources can be more efficient for depression, behavioral therapy directed toward coping effectiveness training could improve quality of life in ALS. Moreover, dyadic interactions should receive more attention, specifically about reciprocal protective buffering behavior that could assist couples in coping with the challenges faced during illness.

## Limitations

The main limitations of the present study are related to the small sample size which over time due to drop outs lead to an inadequate predictors:sample size ratio for regression modeling at the last two time points. The generalizability of the results should be therefore considered with caution. Moreover, some of the used measures showing moderately strong psychometrical characteristics pose limitations. Finally, due to a relative high variability and wide range of the time between the measurement time points (3–6 months), which were unavoidable for clinical and organizational reasons results should be interpreted with caution. In future studies more information about dropouts would be desirable to ensure that not only those who manage to adjust well remain in the sample.

## Conclusions

Despite these limitations we conclude that psychological strategies exist to cope well with ALS—against all odds—and effort should be invested toward improving support instead of further legalizing euthanasia. The here presented data and all previous work on QoL and psychological aspects in ALS from our group (Lulé et al., 2008, 2011, 2014; Matuz et al., 2010; Real et al., 2014) as well as from other groups clearly disprove the widespread prejudice of a negative QoL and high depression in all stages of ALS and strongly support a palliative approach to ALS which equally implies advice with regards to coping strategies, provides reasonable amount of illness and support related information at any one time and heartens patients to seek social support.

### Conflict of interest statement

The authors declare that the research was conducted in the absence of any commercial or financial relationships that could be construed as a potential conflict of interest.

## Abbreviations

ALS: amyotrophic lateral sclerosis
MS: multiple sclerosis
QoL: quality of life
BDI: Beck's Depression Inventory
ADI-12: ALS Depression Inventory
SEIQoL-DW: The Schedule for the Evaluation of Individual Quality of Life Direct Weighting
BSSS: Berlin Social Support Scales
MNDCS: Motor Neuron Disease Coping Scale.
