# Supplementary material for: Psychosocial adjustment to ALS: a longitudinal study
Source: Front Psychol. 2015 Sep 14;6:1197. doi: 10.3389/fpsyg.2015.01197 (PMC4568392; doi:10.3389/fpsyg.2015.01197)
Supplement: Supplementary file 1 [file Table1.DOCX]

| **Indicators of Psychosocial Adjustment** | **Measurement** | **Artificial Ventialtion*** | **Artificial Nutrition**** | **Education***** | **Gender** | **Illness Duration****** |
| --- | --- | --- | --- | --- | --- | --- |
| ADI-12 | T1 | *U* = 82.5  p = 0.8 | *U* = 47.5  p = 0.3 | *U* = 68  p = 0.2 | *U* = 69.5  p = 0.3 | *U* = 65  p = 0.2 |
|  | T2 | *U* = 47  p = 0.4 | *U* = 39.5  p = 0.2 | *U* = 33  p = 0.07 | *U* = 59.5  p = 0.9 | *U* = 35.5  p = 0.1 |
|  | T3 | *U* = 23.5  p = 0.9 | *U* = 23.5  p = 0.09 | ***U* = 21**  **p = 0.05** | *U* = 53  p = 0.3 | *U* = 26.5  p = 0.1 |
|  | T4 | *U* = 18.5  p = 0.3 | *U* = 15.5  p = 0.09 | *U* = 13.5  p = 0.07 | *U* = 41  p = 0.2 | *U* = 20  p = 0.2 |
| SEIQoL | T1 | *U* = 73  p = 0.4 | *U* = 57  p = 0.7 | *U* = 56  p = 0.09 | *U* = 122.5  p = 0.1 | *U* = 116.5  p = 0.2 |
|  | T2 | *U* = 53.5  p = 0.9 | *U* = 38.5  p = 0.3 | *U* = 52  p = 0.9 | *U* = 66.5  p = 0.4 | *U* = 66.5  p = 0.3 |
|  | T3 | *U* = 31  p = 0.8 | *U* = 31  p = 0.5 | *U* = 25.5  p = 0.2 | *U* = 31.5  p = 0.6 | *U* = 54  p = 0.2 |
|  | T4 | ***U* = 9.5**  **p = 0.05** | *U* = 22  p = 0.5 | *U* = 25  p = 0.1 | *U* = 36.5  p = 0.2 | *U* = 34  p = 0.5 |

*Patients grouped based on their respiratory method: 1.with artificial ventilation 2. Without artificial ventilation

**Patients grouped based on their nutrition method: 1. With PEG and 2. Without PEG

***Patients grouped based on their education level: 1. Low (10 or less years of study) and 2. Higher (more than 10 years of study)

****Patients grouped based on the duration of their illness in months (median split): 1. < 36 month 2. > 36 months
